# Experimental investigation of seismic response of precast concrete panels with castellated keys support pillar connections under in-plane cyclic loading

**DOI:** 10.1038/s41598-023-35386-z

**Published:** 2023-12-08

**Authors:** Zuowei Wang, Jianchang Zhao, Xiangheng Chen, Shengwei Liu, Buzhen Ma

**Affiliations:** 1 School of Civil Engineering, Lanzhou Jiaotong University, Lanzhou, 730070 Gansu China; 2 Research Institute, Lanzhou Jiaotong University, Lanzhou, 730070 Gansu China; 3 Tianshui Architecture Design Institute, Tianshui, 741007 Gansu China

**Keywords:** Civil engineering, Engineering

## Abstract

The reliable joint connection between precast members is vital to resisting earthquake loads in precast concrete structures. To further improve the convenience of precast construction, this paper presents a new horizontal joint of precast concrete panels with castellated keys supports pillar connections based on the stable mechanical properties of the wet concrete joint. To examine the seismic response of this connection type, a series of in-plane cyclic loading tests were carried out using six full-size precast specimens and one cast-in-place specimen for comparison. The influences of axial compression ratio, joint width, and joint concrete strength on the seismic indicators of the precast concrete panel were considered in the design of specimens. The test results showed all specimens had the same damage process, and the ultimate failure modes combined compression and bending. The precast specimens exhibited similar seismic performance to the cast-in-place specimen, especially whose joint concrete strength is higher than the precast concrete panel. Based on the load–displacement test curve, a hysteretic curve model that included both the envelope curve and the stiffness degradation law was proposed. The predictions from the model showed good compatibility with the experimental results, and the model can be used as a reference for analyzing the elastic–plastic response of the precast concrete panels with castellated keys supporting pillar connections.

## Introduction

Since the twenty-first century, precast building, including the advantages of energy saving, environmental protection, high construction efficiency, and better quality control^1^, has become a new wave of the development of building industrialization in China^2,3^. Owing to the considerable stiffness against lateral forces, reinforced concrete shear wall structures have a significant market share in high-rise buildings, and research on their prefabrication has gained popularity in many countries^4–8^. From the previous earthquake disaster phenomena and research results, the high-reliability joint connection between precast concrete panels is a critical foundation ensuring the precast structure system's integrity and seismic performance^9,10^. The horizontal joint connection between the precast wall panels plays an essential role in load transfer since precast concrete shear wall structures would eventually experience compression-bending or shear-bending failure under horizontal seismic loading.

Existing research on precast concrete wall panels' horizontal joint connection forms is more beneficial and diverse^11^. The joint type can be generally divided into wet and dry joints from the construction operation. Post-tensioned prestressed joints, bolt joints, keyed dry joints, and other types of dry joints are currently prevalent^3,12,13^. The dry joint has the advantages of convenient assembly operation, high construction efficiency, and better repairability after an earthquake; however, the technology is more complex, and the construction cost is higher than wet joints^7^. In addition, the failure mode of precast panels with the dry joint differs from that of cast-in-place panels regarding mechanical performance^14^, and the damage of precast members after the earthquake is more likely to be concentrated in the joint area or the additional components for connection^12,15,16^. Precast panels with wet joints, which have more stable mechanical properties, on the other hand, have crack development and final failure modes that are comparable to those of cast-in-place panels.

The connection of vertical steel bars in the precast concrete panels using horizontal wet joints is generally carried out by partial wet construction in the field, including the grouted duct connection, the mechanical connection, the reinforcing bar overlap connection, etc.^4,17,18^. Regardless of the objective of those horizontal wet joint connections is to achieve performance similar to that of cast-in-place buildings, there are still issues with connection reliability, a convenient construction method, and construction costs in precast concrete panels. The construction quality of actual precast concrete panel structures is unsatisfactory due to several issues^19,20^ , including the difficulty of assembling precast members, poor casting quality in horizontal cast-in-place belts, inability to continue construction due to concrete curing in the cast-in-place part, unstable mechanical properties caused by complex technical requirements for construction in grouted duct connections, and etc.^7^.

The current research on precast concrete structures should consider all relevant elements, including construction operability, construction time, and economic cost, in addition to ensuring the reliability of joint connections in terms of mechanical properties^21,22^. In light of the issues raised in earlier research, this work presents a horizontal joint type that may be assembled quickly and cost-effectively, combined by a precast support pillar with castellated keys and a technique for U-shaped vertical reinforcing bar loop overlap—Precast concrete panels with castellated keys support pillar connections. To validate the seismic response of the precast panels when using the abovementioned connections in the panel-foundation horizontal joint, six full-size precast concrete wall panels and one cast-in-place wall panel were tested under in-plane cyclic loading. The seismic performance indexes were discussed, including crack patterns, failure modes, load–displacement curve characteristics, stiffness degradation, and energy dissipation capacity. A hysteretic model was formulated based on the experimental load–displacement curve to provide a theoretical reference for the seismic response prediction and seismic research of precast concrete panels with castellated keys support pillar connections. The results can be employed to develop a precast concrete panel joint connection system and provide a concept for researching and promoting economic and reliable joint connections.

## Structural characteristics

**Figure 1:**
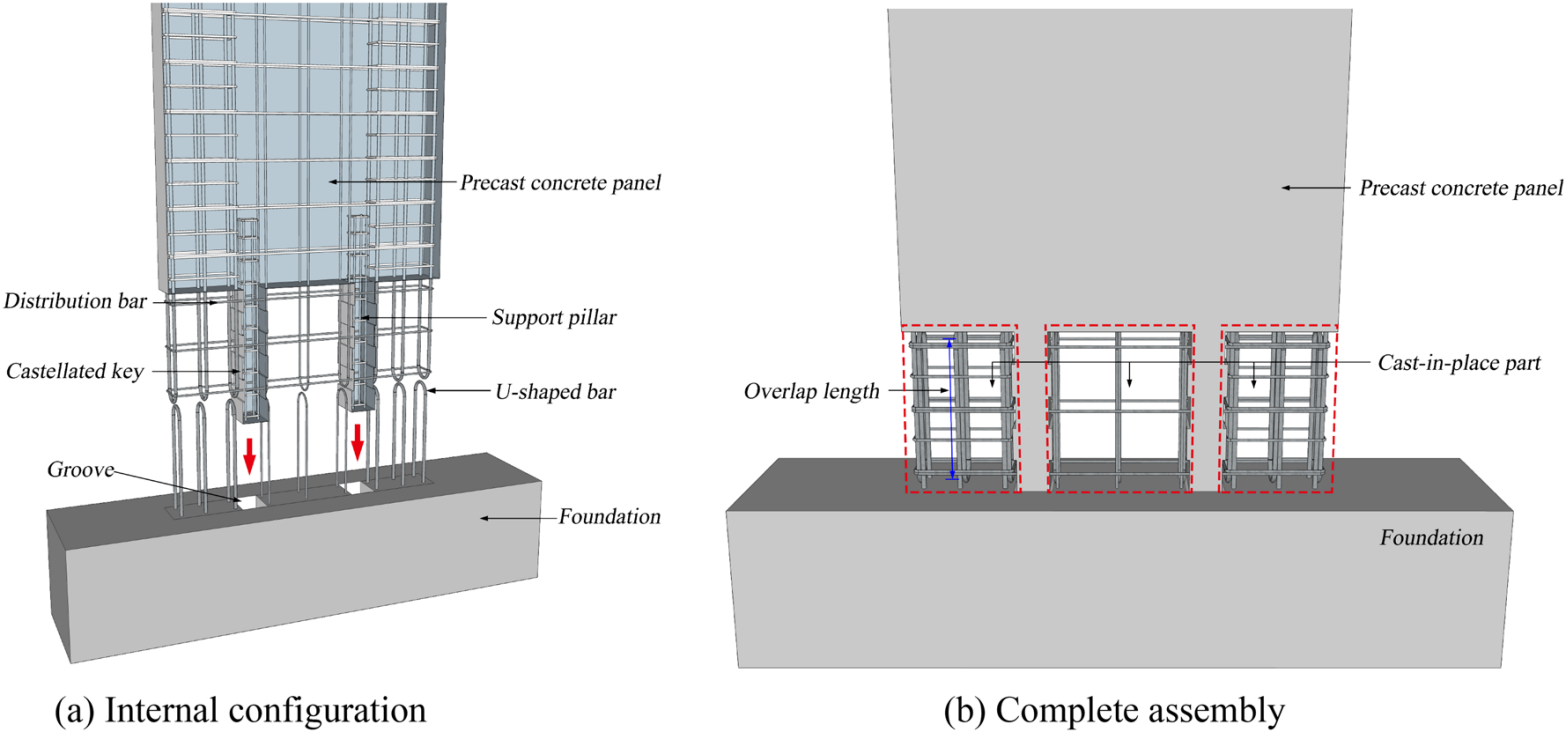
Horizontal joint configuration.

The horizontal joint system of precast concrete panels with castellated keys support pillar connections consisted of the following parts: an upper precast concrete panel, lower foundation (lower precast concrete panel), support pillars with castellated keys, vertical U-shaped steel bars, and horizontal distribution steel bars. As shown in Fig. 1a, during assembly, the upper precast panel, which was prefabricated with castellated key support pillars, can be inserted into the grooves of the lower precast panel corresponding to the pillars. The shear capacity of the interface between the precast member and the cast-in-place area can be enhanced by utilizing castellated keys on the support pillars^23^. The continuous construction of the precast panel of the next floor can be accomplished after the assembly of the current panel without the necessity of waiting for the cast-in-place concrete to harden to reach the target strength because the support pillars, which are equipped with structural reinforcement independently, and can bear the vertical load of the upper precast members well. The docking process of precast members at the construction site was more convenient based on the positioning of the grooves. And since the support pillar bore the upper construction load, the assembly efficiency was greatly improved. The support pillar was positioned between the vertical distribution bars of the upper precast panel and did not influence the connection of any vertical reinforcing bars. A U-shaped bar overlap that was easy to construct and consistently effective was used to connect all vertical reinforcing bars. Horizontal distribution bars in the horizontal joint region were prefabricated in the support pillars of the upper precast panel and were.bound to the vertical reinforcing bars after the assembly colligates, as illustrated in Fig. 1b.

**Figure 2:**
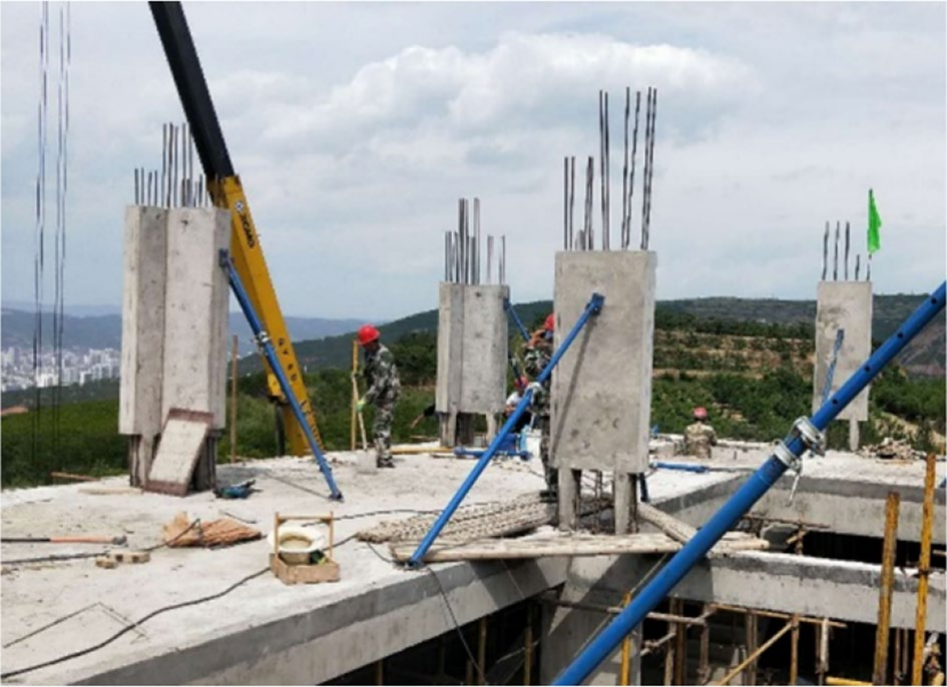
Pilot project using precast concrete panels with castellated keys support pillar connections.

The typical cast-in-place region was divided into various sections by the support pillars; thus, the concrete in the central cast-in-place region was constrained in two dimensions by the precast panel and pillar. This connection can reduce the shear dislocation at the overall horizontal interface between the old and new concrete due to the segmented and discontinuous state of the interface. In addition, the support pillar with independent reinforcement enhances the shear capacity of the horizontal joint. The bottom corner of the panel, an important area where the precast panel was seriously damaged under horizontal seismic loading, was strengthened by cast-in-place to have similar damage and energy dissipation to the cast-in-place panel in the process of structural failure. Figure 2 demonstrates a pilot project using precast concrete panels with castellated keys support pillar connections for a multi-storey residential building. Based on the problems encountered in the pilot project, the seismic performance of the system was studied experimentally by low-cycle loading to ensure the reliability of the precast concrete panels in the current study.

## Experimental program

### Test specimen design

**Figure 3:**
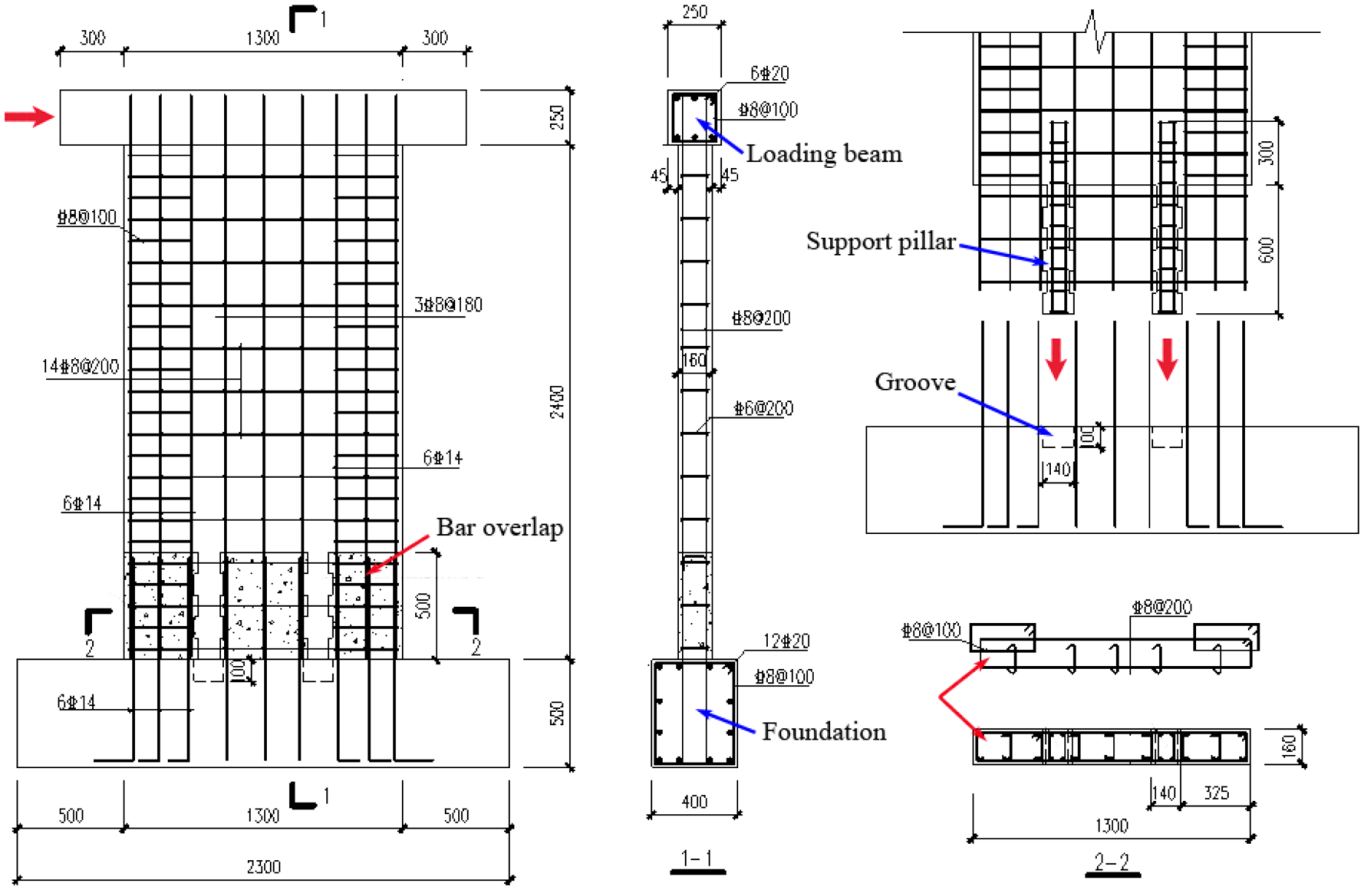
Reinforcement details of standard precast specimen PSW-1.

To reflect the seismic performance of precast members directly and to show the assembly process clearly, seven full-scale specimens were designed and constructed. Test specimens consist of six precast concrete panels with castellated keys support pillar connections (referred to as precast panel specimens) and one cast-in-place concrete panel specimen. All test specimens were composed of a test wall panel, top loading beam, and bottom foundation beam. The cross-section of the panels was rectangular, with a length of 1.3 m and a width of 0.16 m. The test panel was a common concrete shear wall, the first level shear wall with a length–width cross-section ratio greater than 8 under GB 50011–2010^24^. The height of the test panel was 2.4 m, and the calculated height H of the horizontal load was 2.525 mm. The geometry of the test specimens is presented in Fig. 3.

In the related test, the influencing factors, including axial compression ratio, horizontal joint width, and joint concrete compressive strength, were investigated. The configuration of the specimens is presented in Table 1. To prevent a brittle failure mode.of the specimen under eccentric compression, the limit value of the axial compression ratio *μ* was not greater than 0.5 in accordance with JGJ 3–2010^25^. The axial compression ratio *μ* of the cast-in-place specimen SW-1 and the standard precast concrete specimen PSW-1 was designed to be 0.2. The axial compression ratio *μ* of the comparison specimen PSW-3 and PSW-4 were 0.1 and 0.3, respectively. The formula for the vertical load *N*_*t*_ is given as follows:

1 $$N_{t} = \frac{{\mu Af_{c} }}{{{\upgamma }_{G} }}$$

where *μ* depicts the axial compression ratio, *A* is the cross-section area, and *f*_*c*_ is the concrete compressive strength; ${\upgamma }_{G}$ represents the gravity load amplification factor considering seismic, which was set to 1.2 according to JGJ 3–2010^25^. The horizontal joint width *l*_*w*_ of the standard precast concrete specimen was designed to be 0.5 m, while the *l*_*w*_ of the comparison specimen PSW-4 and PSW-5 were 0.4 m and 0.3 m, respectively. The minimum grade of concrete compressive strength is C25 in accordance with GB 50011–2010^24^ when the yield strength of the steel bar is greater than 400 MPa. The concrete compressive strength grade in the precast panel and the joint cast-in-place area was selected to be C25 in the test, except that the grade in the joint cast-in-place area of comparison specimen PSW-6 was C30.

**Table 1:** Configuration of specimens.

Specimen number	Joint width, *l*_*w*_ (mm)	Axial compression ratio, *μ*	Vertical load, *N*_*t*_ (KN)	Concrete class of Joint
SW-1	–	0.2	412	–
PSW-1	500	0.2	412	C25
PSW-2	500	0.1	206	C25
PSW-3	500	0.3	618	C25
PSW-4	400	0.2	412	C25
PSW-5	300	0.2	412	C25
PSW-6	500	0.2	412	C30

Concrete compressive strength grade of the precast panel is C25.

### Material properties

The materials used for the test specimens include C25 and C30 grade concrete for the precast panel and the joint cast-in-place area; HRB400 grade steel bar with diameters of 8 mm and 14 mm (hot-rolled rebar bar: yield strength greater than 400 MPa). Mechanical tests were conducted on the concrete per the instructions described in GB/T 50081–2019^26^ and steel bar per the instructions described in GB/T 228.1–2010^27^. The physical properties of materials are summarized in Table 2.

**Table 2:** Material physical properties.

Concrete	Grade	Failure load, (kN)	Compressive strength, *f*_*cu*_ (MPa)	
	C25	516.7	23.0	
	C30	653.3	29.0	
HRB400 steel bar	Diameter, *d* (mm)	Yield strength, *σ*_*y*_ (MPa)	Ultimate strength, *σ*_*u*_ (MPa)	Elongation, (%)
	8	418.0	641.9	24
	14	451.7	649.9	22

### Specimen making and connection details

For the seismic concrete wall panels, the dimensions and main reinforcement details of all specimens are in accordance with the requirements of GB 50011–2010^24^. The distribution bars of wall panels were arranged in double rows; the spacing between the vertical distribution bars was 180 mm, while it was 200 mm for the horizontal distribution bars. Six vertical reinforcement bars with a diameter of 14 mm and *Φ*8@100 stirrups were allocated in the edge member with a higher reinforcement ratio.

At the bottom of the panel, the horizontal joint height for the PSW-1 precast standard specimen was 500 mm. All vertical reinforcing bars were connected by a U-shaped loop bar overlap at the full joint height. Two support pillars with a cross-section of 100 mm × 160 mm, which were equipped with four 8 mm vertical reinforcing bars and *Φ*8@100 stirrups, were arranged between the vertical distribution bars of the upper precast panel as illustrated in Fig. 3. The castellated keys on the support pillar were arranged at the interface between the pillar and the cast-in-place part. Each key was 20 mm in height and 100 mm in width.

The lower panel (the bottom foundation in the test) was provided with a connecting groove with a depth of 100 mm for the upper support pillar. PSW-2, PSW-3, and PSW-6, identical to PSW-1, were used to investigate the influence of axial compression ratio and joint concrete strength. PSW-4 and PSW-5 were identical to PSW-1 except for the height of the support height, which was to investigate the effect of horizontal joint height. The dimensions and main reinforcement configurations of the cast-in-place reference specimen SW-1 without horizontal joint were the same as those of the standard precast specimen PSW-1. The wall panel, top loading beam, and bottom foundation beam were cast in whole. The vertical reinforcing bars of all specimens were anchored on the top loading beam or the bottom foundation beam to ensure load transfer effectively.

**Figure 4:**
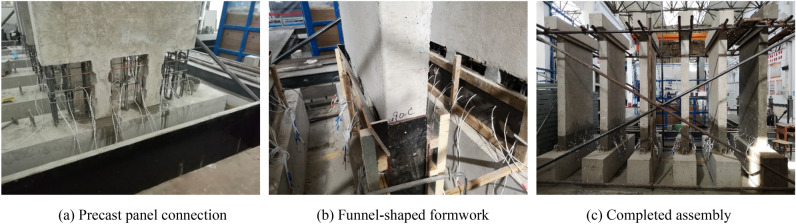
Specimen fabrication and assembly.

The fabrication and assembly of specimens are shown in Fig. 4. After standard concrete curing of the precast member, the concrete surface of the precast member at the interface between the precast part and cast-in-place area was chiseled to ensure roughness, and then the precast member was hoisted and connected to the groove of the bottom foundation via the support pillar conveniently (a small amount of high strength mortar was added to the groove before connecting). After completion of the abovementioned processes (Fig. 4a), the stirrups were placed in the edge member of the cast-in-place part, and the formworks were installed for concreting. A height of 50 mm was left at the top of the cast-in-place section to set a funnel-shaped formwork, as shown in Fig. 4b, since full concreting at the upper interface of the cast-in-place part during construction was challenging. The upper edge of the funnel-shaped formwork was 50 mm higher than the cast-in-place part. To ensure that the upper interface of the cast-in-place part was densely filled, the entire funnel-shaped formwork was filled with concrete and vibrated. Then, the funnel-shaped formwork was removed, and the excess concrete at the funnel was cut within 3 to 6 h after concreting (the concrete was in the initial setting but not hardening completely). The rest of the formwork would be retained until the concrete curing was completed.

### Test setup and loading scheme

**Figure 5:**
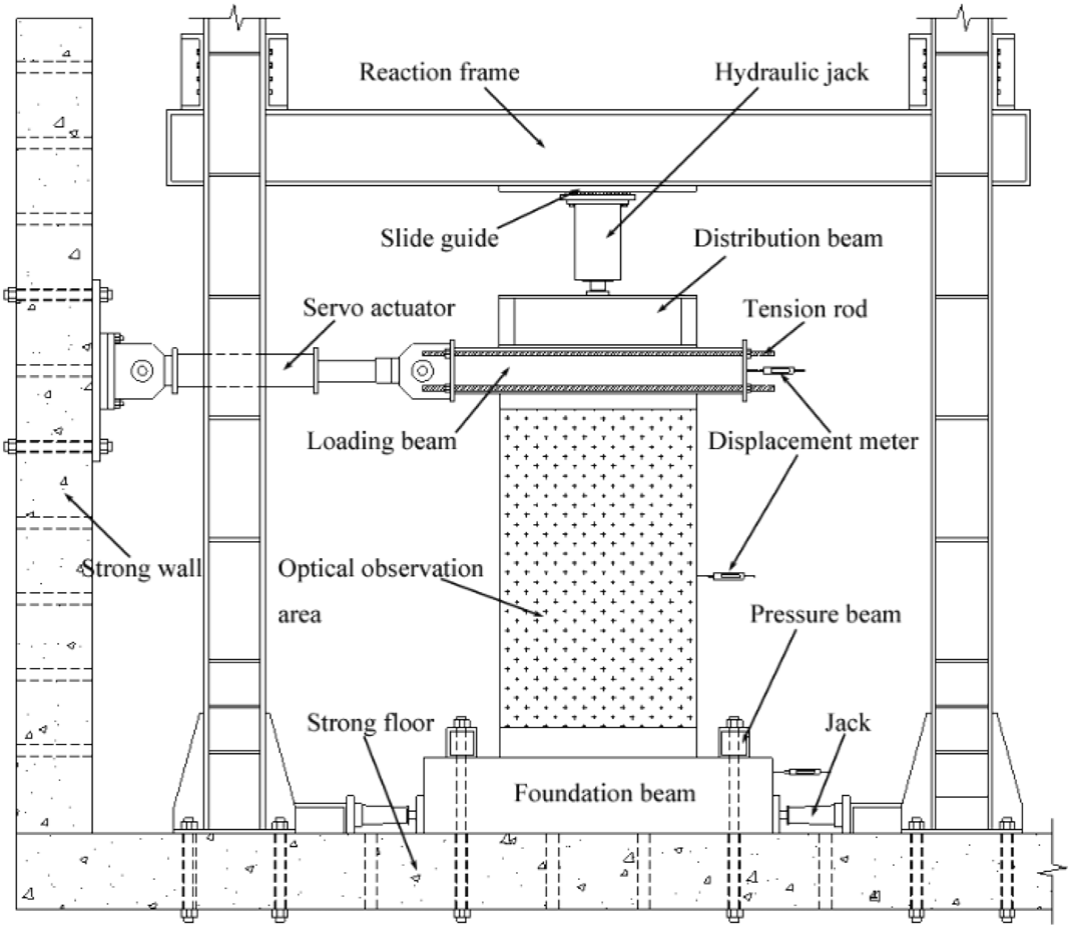
Schematic test setup.

**Figure 6:**
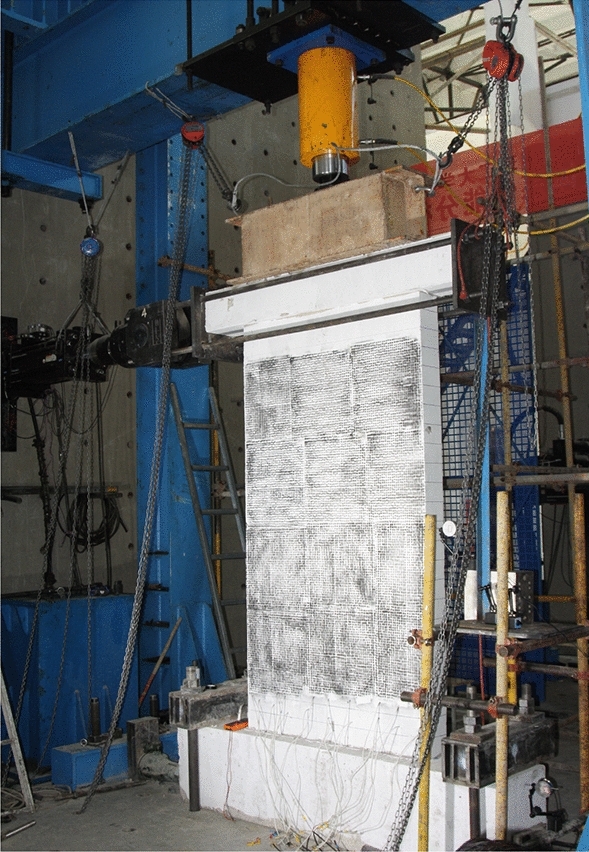
Photo after installation.

**Figure 7:**
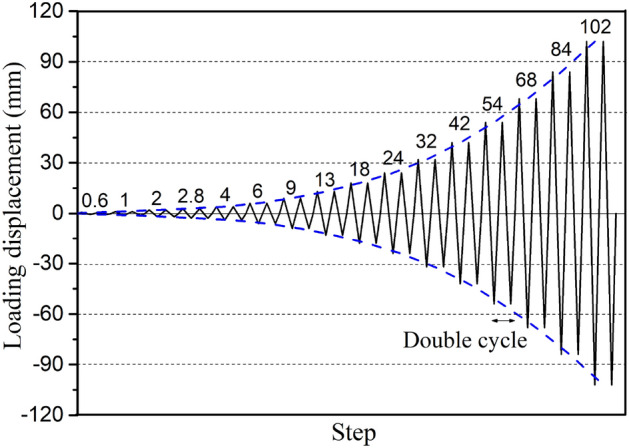
Loading history.

The test loading system was designed according to the mechanical characteristics of the single-piece cantilever wall and the test requirements of JGJ/T 101–2015^28^. The test setup mainly consisted of a strong floor, strong wall, reaction frame, and vertical-horizontal loading device. The test setup details are shown in Figs. 5 and 6. The horizontal cyclic load was applied to the loading beam through the strong wall, electro-hydraulic servo system, and connecting tension rods. The hydraulic servo actuator's two ends were hinged; thus, the rotation of the upper panel in the plane was not constrained. The total number of horizontal loading cyclic steps was equal to 16, and double cycles were used for each step used, as demonstrated in Fig. 7. The test was finished when the horizontal load dropped to 85% of the peak load or when the sample was damaged severely to continue to load. The horizontal force and displacement at the top loading point were measured by the hydraulic servo actuator during the test, and the displacements at the other critical points were measured by LVDT displacement meters and XTDIC-CONST optical strain measurement system. The single surface of the panel was observed through the optical system in this test, and the sampling frequency employed was 1 Hz. The data of displacement meters were collected by DH5922 dynamic strain measurement and the sampling frequency employed was 5 Hz.

## Experimental results

### Typical crack and failure modes

**Figure 8:**
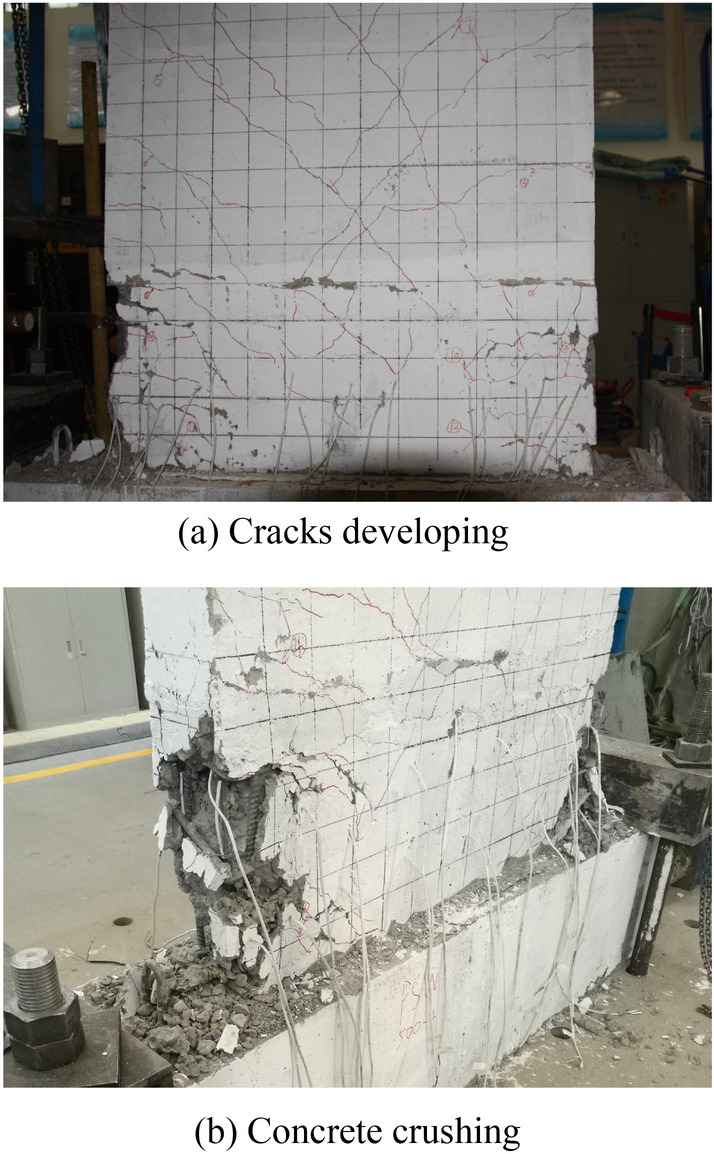
Typical damage.

According to the findings, all specimens had similar failure processes and showed typical compression and bending failure modes. In general, the horizontal bending cracks at the early loading stage (reaching 25–35% of the peak load) appeared successively at the bottom of the specimen (less than 1/3 panel height) and gradually developed into symmetrical diagonal cracks. The significant proportion of the cracks extended horizontally 100 mm–200 mm from both sides of the panel to the middle and then propagated diagonally downward at 30° ~ 45°, as illustrated in Fig. 8a (the higher position of the cracks in the panel, the greater angle to the ground). When the load reached 80–90% of the peak load, the main symmetrically crossed cracks no longer increased, and the concrete at the bottom corners appeared slightly crushing. The panel corner exhibited greater concrete peeling from the peak load until the end of the test, exposing the reinforcing bars, which were perceived as having significant yield deformation (Fig. 8b).

**Figure 9:**
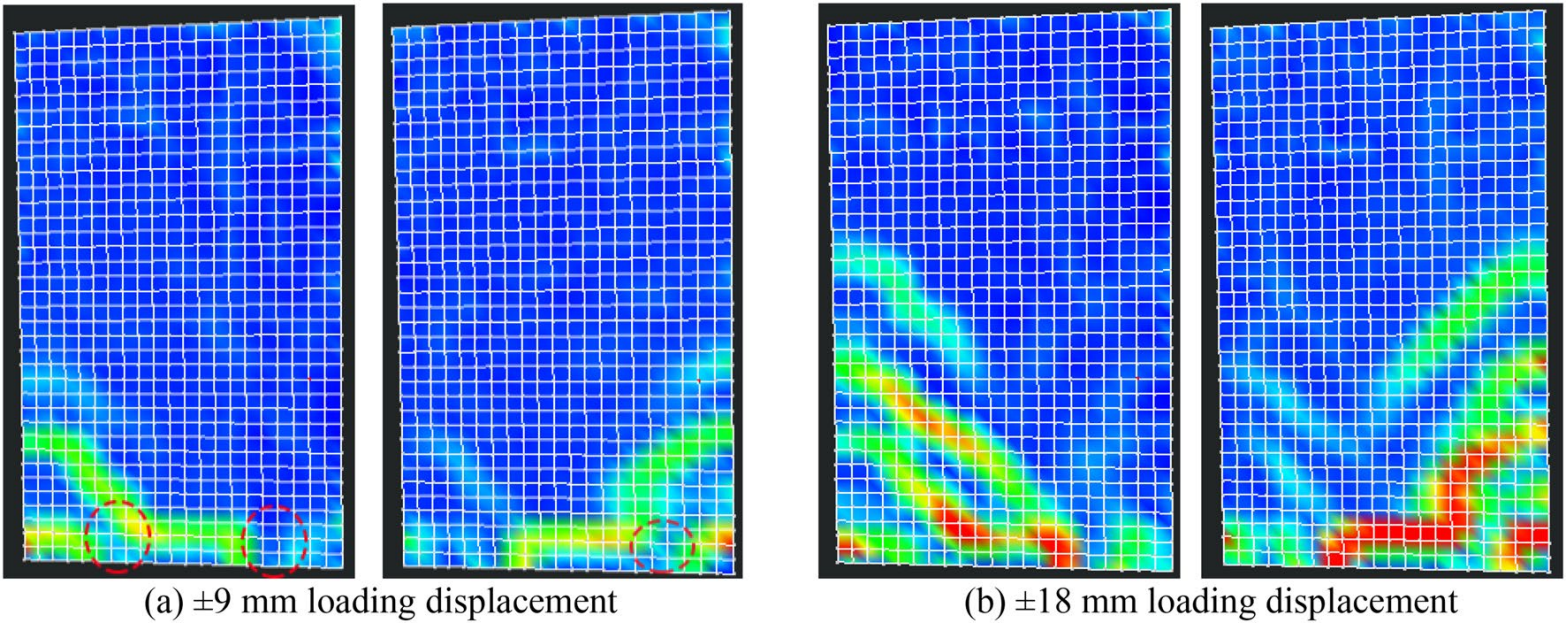
Optical observation for PSW-1.

**Figure 10:**
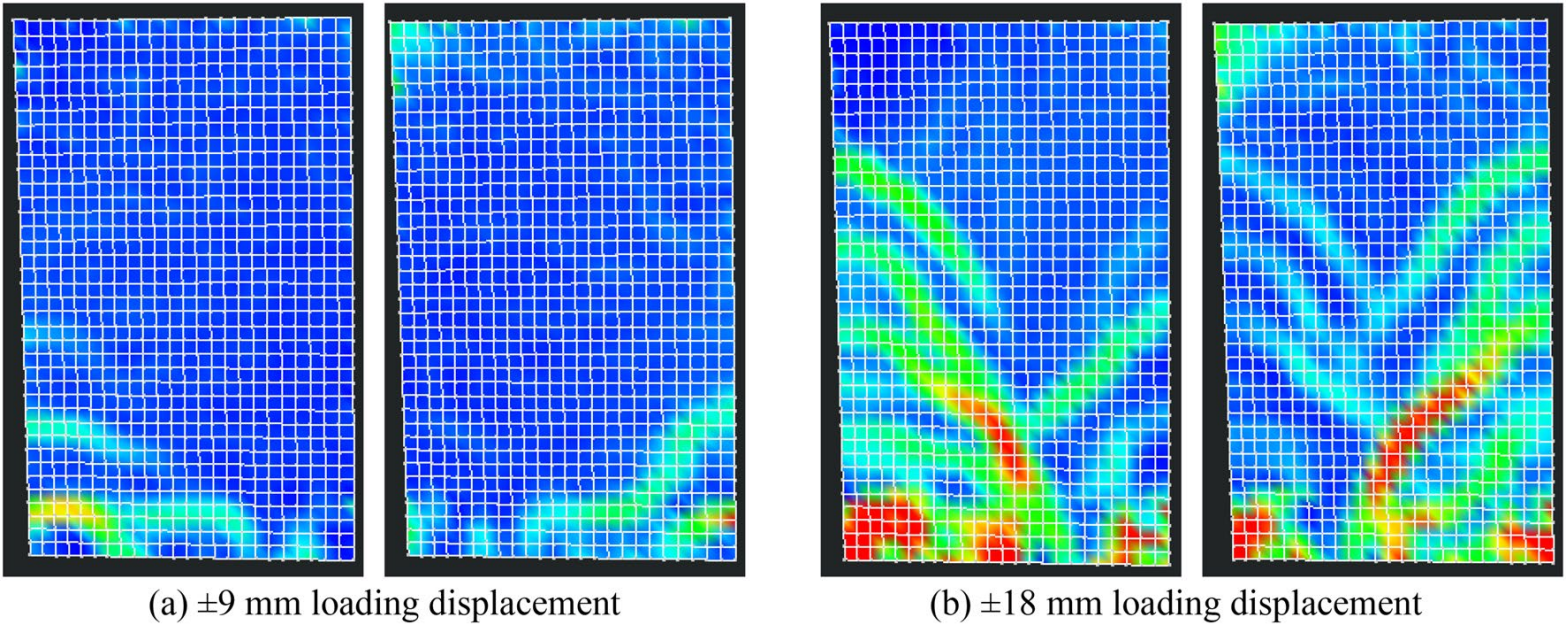
Optical observation for PSW-3.

In the early loading stage, the precast specimens' horizontal cracks typically emerged first at the upper interface of the cast-in-place part in the horizontal joint, compared to the damage process of the cast-in-place specimen SW-1. The interface between the precast specimen's old and new concrete was weak, and the cracking load of the precast specimen was less than that of the cast-in-place specimen. It was noteworthy that the precast specimen PSW-6 with higher concrete strength for the cast-in-place part had improved the above weak interface. The cracks at the weak interface in PSW-6 appeared almost simultaneously with other horizontal cracks. As the precast specimen was supported with the castellated keys support pillars at the horizontal joint, cracks at the weak interface could not propagate through the entire panel cross-section. Some diagonal cracks extended to the cast-in-place part and merged with the interface cracks, as shown in Figs. 9 and 10.

**Figure 11:**
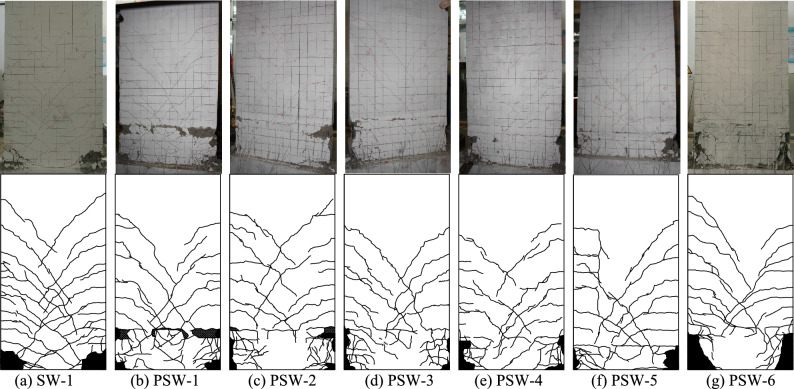
Final failure of all specimens.

From the view of the final damage (Fig. 11), the concrete crushing area at the bottom corner of the cast-in-place specimen was smaller than that of the precast specimen, whose height of the crushing area was usually the whole horizontal joint height. It was related that the vertical reinforcement connection at the horizontal joint used U-shaped bar overlap splice, and the overlap length was the horizontal joint height. While the joint section was under tension, in addition to the bond stress between the lapped bar and the concrete, the vertical reinforcement applied pressure to the core concrete in the lap area via a U-shaped bar loop to ensure the transfer of tensile stress. Under cyclic load, the concrete at the bottom corner of the precast specimen was not only damaged from the compression zone but also subjected to compression damage from the U-shaped bar loop under the tension zone. Therefore, the alternating action of the two states increased the concrete crushing area at the bottom corner of the precast specimen (whole horizontal joint height).

### Hysteresis curves

**Figure 12:**
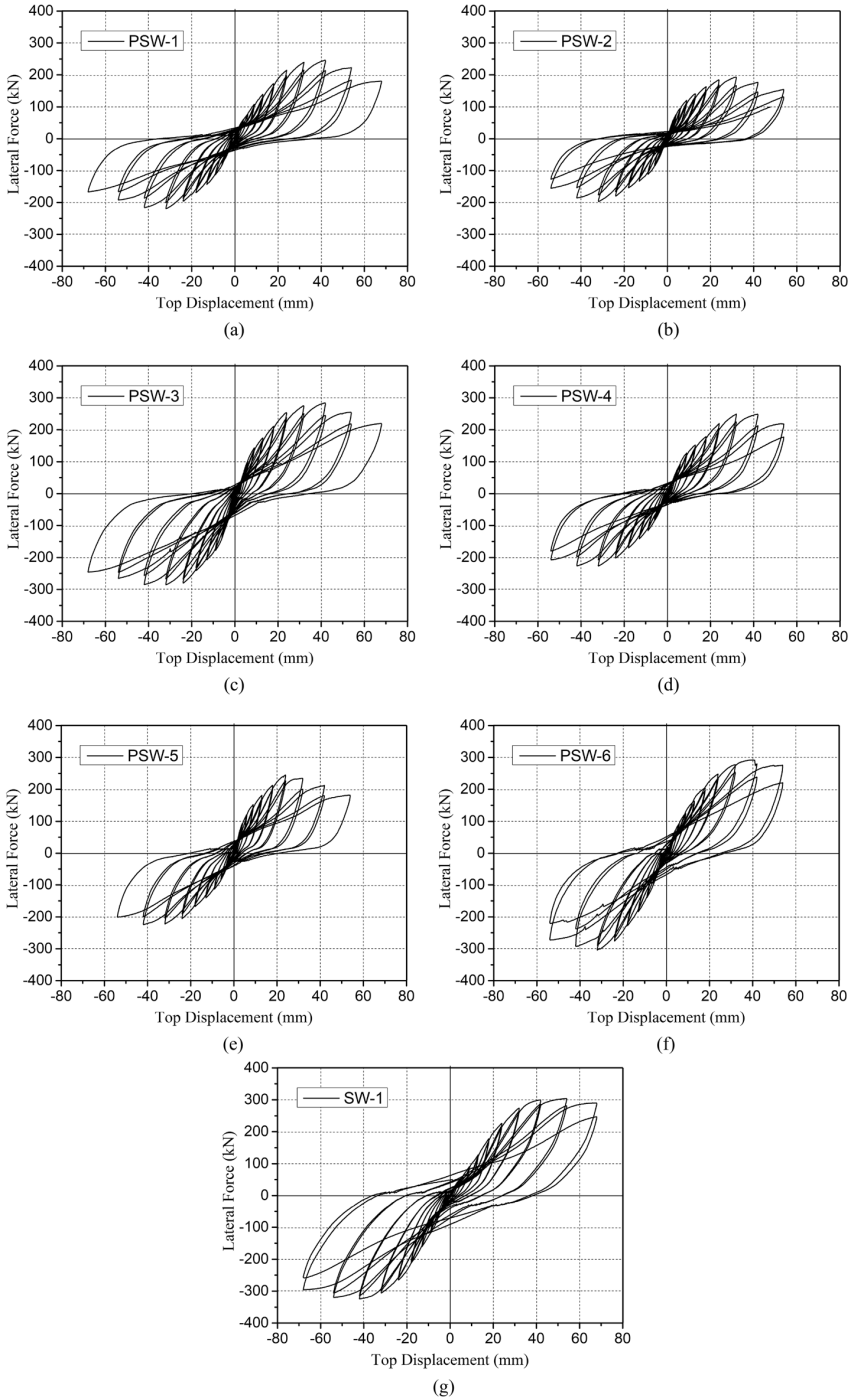
Hysteretic curve of test specimen: (**a**) PSW-1; (**b**) PSW-2; (**c**) PSW-3; (**d**) PSW-4; (**e**) PSW-5; (**f**) PSW-6; (**g**) SW-1.

The hysteretic curves of the test can be plotted by the lateral force versus top displacement, as shown in Fig. 12. According to the curves, it can be stated that the shape and trend of the hysteretic curves of all specimens were similar with the following characteristics: (a) The load–displacement curve changed linearly in the initial stage. The initial stiffness degradation was not obvious, and almost no residual deformation occurred after unloading; (b) After the panel cracked, the ascending portion of the hysteretic curve showed a reduced slope, and the curve had a nonlinear growth trend. At this point, the continuous accumulation of residual deformation resulted in the gradual increase of the enclosed area of the hysteretic loop (increasing energy consumption); (c) After reaching the peak of the curve, the bearing.capacity of the specimen began to decrease gently; however, there was no significant sudden drop. The hysteretic curve's descending portion exhibited a more significant curvature, demonstrating effective energy dissipation.

When the diagonal cracks in the panel were fully developed (near the peak load), the hysteresis loop would appear as a pinching phenomenon. The reason was that the main diagonal cracks in the panel no longer increased in number but increased in width because of a more significant slip between reinforcement and concrete. Due to the closure of the crack produced by the last cycle, the load's enhanced efficiency in the ascending part of the curve decreased with increasing horizontal displacement. Therefore, the middle part of the hysteresis loop appeared as a pinching phenomenon which became more obvious with the further increase of the horizontal displacement. When the horizontal load reached the peak, the precast specimen's bearing capacity declined more rapidly, and the pinching phenomenon associated with that is more evident than it is for the cast-in-place specimen SW-1. However, the precast specimen's hysteretic curve had a fuller descending part, indicating that the energy dissipation was successful. Additionally, the specimen PSW-6's pinching phenomenon was obviously improved due to the enhanced joint concrete strength.

### Envelope curves and characteristic parameters

**Figure 13:**
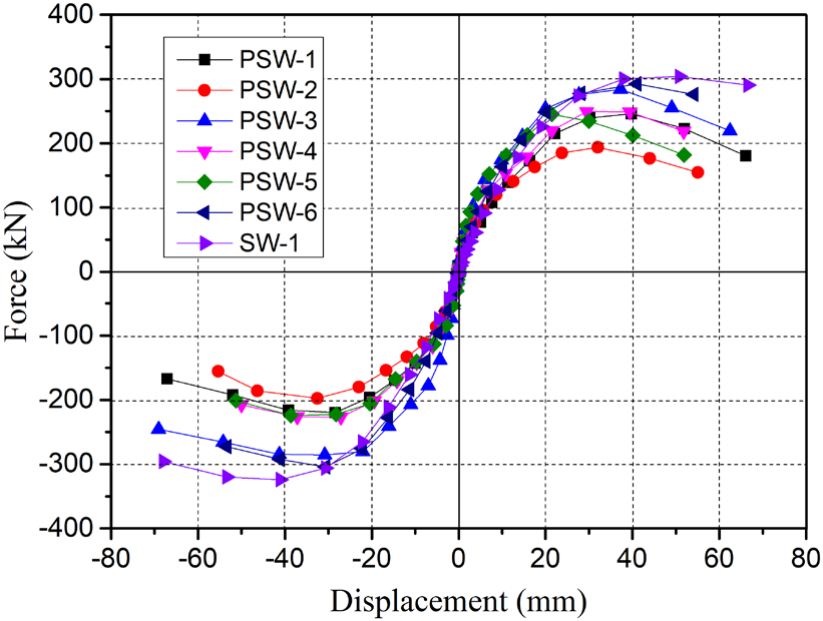
Envelope curves.

The Fig. 13 demonstrates the envelope curves obtained from the peak points of each cycle of hysteresis curves. All curves with similar patterns and characteristics can be separated into three segments, namely an elastic segment, a nonlinear rising segment, and a degraded segment, as shown in Fig. 13. In the elastic segment, the specimen was almost intact and showed no noticeable stiffness degradation. As the horizontal displacement increased, the envelope curve showed a nonlinear rising trend (after the specimen cracks); however, there was no characteristic intuitive point indicating the yield state before the peak of the curve. The specimen's bearing capacity progressively declined without experiencing a significant decline once the horizontal load peaked.

**Figure 14:**
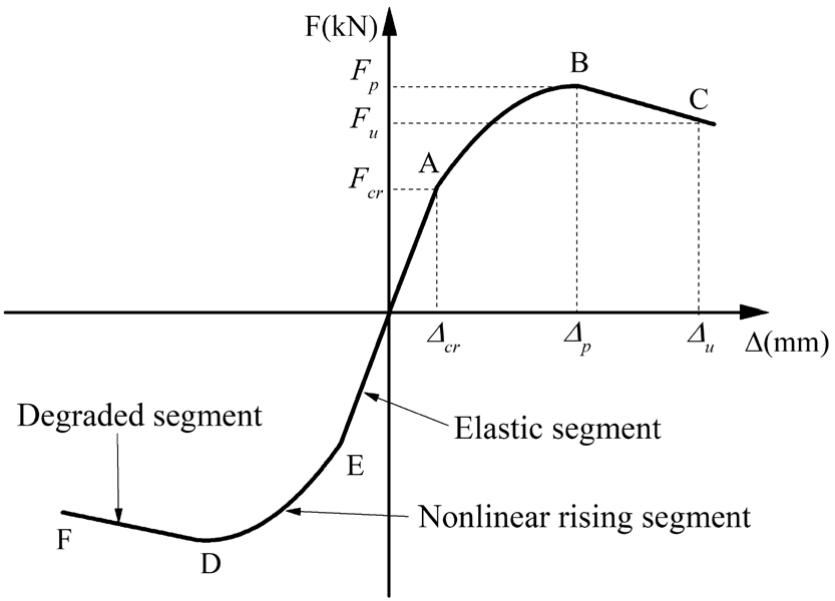
Three-segment model of envelope curve.

According to the characteristics of the envelope curve and the failure process of the specimen, the main characteristic points of the specimen were defined, with a cracking point, a peak point, and an ultimate point, as shown in points A, B, and C in Fig. 14. The crack point was determined by the appearance of the first crack through artificial observation; the peak point was the characteristic point of the maximum horizontal load in the loading process; the ultimate point was taken as the point of 90% peak load in the degraded segment of the envelope curve. To further analyze the wall ductility, the yield point was defined in the nonlinear rising segment of the envelope curve. Because there was no evident inflection point in the rising part of the envelope curve, the yield point was computed using FEMA273 guidelines^20,29^. The load and corresponding displacement of each characteristic point were defined as the characteristic parameters. Table 3 shows the mean values of the cyclic loading test's characteristic parameters.

**Table 3:** Summary of test results.

Specimen number	*F*_*cr*_ (kN)	${\Delta }_{cr}$(mm)	${\theta }_{cr}$(%)	*F*_*y*_ (kN)	${\Delta }_{y}$(mm)	${\theta }_{y}$(%)	*F*_*p*_ (kN)	${\Delta }_{p}$ (mm)	${\theta }_{p}$ (%)	*F*_*u*_ (kN)	${\Delta }_{u}$ (mm)	${\theta }_{u}$ (%)	$\mu$
SW-1	93.6	5.88	1/429	274.7	26.04	1/97	314.1	45.95	1/55	293.1	67.28	1/38	2.58
PSW-1	79.1	4.38	1/577	203.5	21.21	1/119	232.9	39.45	1/64	209.6	50.75	1/50	2.39
PSW-2	69.1	3.44	1/734	167.3	19.25	1/131	195.6	32.25	1/78	176.0	46.97	1/54	2.44
PSW-3	82.1	2.13	1/1185	247.5	18.00	1/140	284.4	39.29	1/64	256.4	54.72	1/46	3.04
PSW-4	63.9	2.18	1/1158	210.7	20.66	1/122	237.6	28.30	1/89	216.0	49.68	1/51	2.40
PSW-5	70.2	1.88	1/1343	204.1	17.32	1/146	234.7	24.83	1/102	211.3	43.56	1/58	2.51
PSW-6	94.7	4.70	1/537	263.2	21.72	1/116	298.3	39.55	1/64	274.8	53.41	1/47	2.46

(1) *F*_*cr*_, ${\Delta }_{cr}$ and ${\theta }_{cr}$ are the cracking load, corresponding displacement, and displacement angle, respectively; *F*_*y*_, ${\Delta }_{y}$ and ${\theta }_{y}$ are the yield load, corresponding displacement, and displacement angle, respectively; *F*_*p*_, ${\Delta }_{p}$ and ${\theta }_{p}$ are the peak load, corresponding displacement, and displacement angle, respectively; *F*_*u*_, ${\Delta }_{u}$ and ${\theta }_{u}$ are the ultimate load, corresponding displacement and displacement angle, respectively; $\mu$ represents the ductility ratio of ${\Delta }_{u}$ to ${\Delta }_{y}$; (2) All parameters are the mean values of absolute values of positive and negative loading.

### Influence analysis of characteristic parameters

#### Cracking load *F*_*cr*_

**Figure 15:**
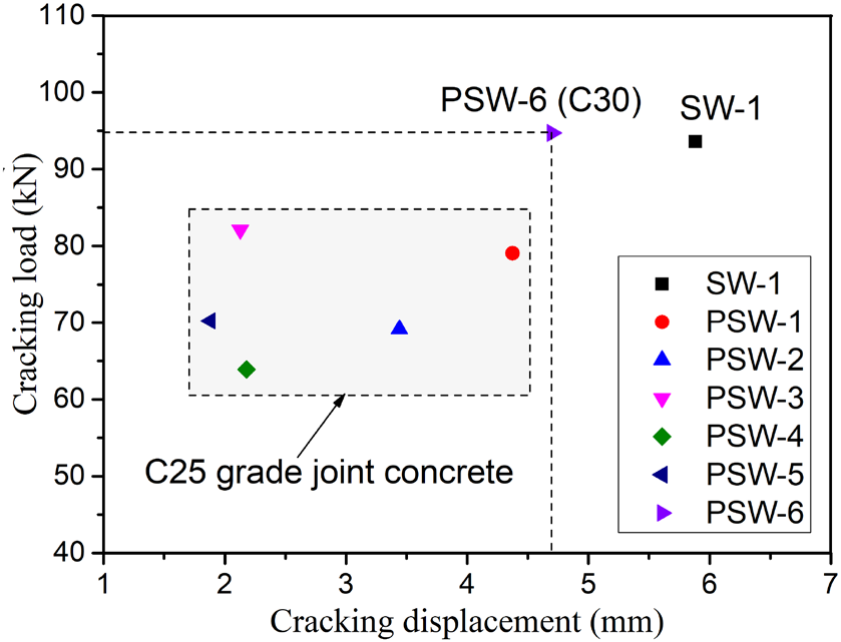
Distribution of cracking load *F*_*cr*_ and corresponding displacement.

The distribution of the cracking load and the corresponding displacement of all samples are shown in Fig. 15. It can be seen from the figure that the cracking load and displacement of precast specimens using C25 grade joint concrete were generally less than those of cast-in-place specimen SW-1. The reason is the fact that the first cracks of precast specimens are more likely to occur at the interface between old and new concrete due to the setting of the cast-in-place part at the bottom. However, as illustrated in Fig. 15, the cracking load of a precast specimen can be clearly raised when the joint concrete strength is improved. It is important to noted that the cracking load of PSW-6 utilizing C30 grade joint concrete was slightly higher than that of cast-in-place specimen SW-1. Increasing the concrete strength of horizontal joint can effectively delay cracking of the weak interface between old and new concrete. Cracking loads of precast specimens with varying axial compression ratio and joint width were generally between 63 and 85 kN for specimens with diverse affecting parameters. The cracking load increased slightly with the axial compression ratio increased (from PSW-2 to PSW-1 to PSW-3, the axial compression ratio increased from small to large). The trend of influence of joint width on cracking load was not evident, however, the cracking load of the specimen with 500 mm joint width (PSW-1) was much greater than that of the specimens with 300 mm (PSW-5) and 400 mm joint widths (PSW-4). This could be due to the fact that the higher interface of new and old concrete is subjected fewer bending moments, and the cracking of the interface appears later. Although the lower interface was subjected to increased bending forces, the crack development at the bottom of the panel might be restrained by the end constraint from the foundation beam (the cracking load of PSW-4 with higher joint width was slightly smaller than that of PSW-5).

#### Peak load *F*_*p*_

**Figure 16:**
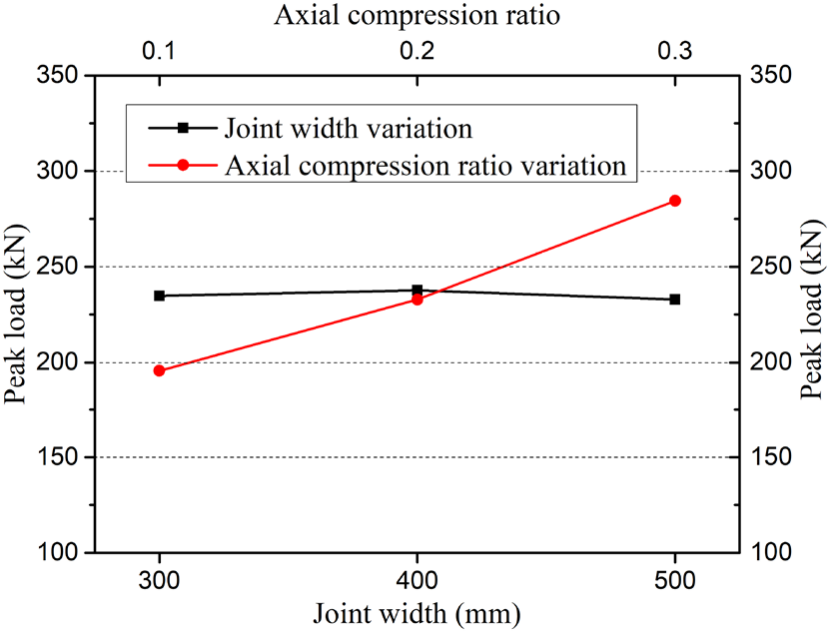
Variation trend of peak load *F*_*p*_ with axial compression ratio or joint width.

The trend of the influences of axial compression ratio and joint width on the peak load is shown in Fig. 16. The peak load of precast specimens was increased significantly with the increase of axial compression ratio from 0.1 to 0.3. Although increasing the axial compression ratio appropriately benefits the structure in terms of horizontal load-bearing capacity, the failure mechanism of the structure may vary under high axial compression ratio, such as brittle failure under small eccentric compression. All specimens in this test demonstrated optimal plastic deformation within the low axial compression ratio range specified by GB 50,011–2010. (The limit value is usually 0.5). In addition, the variation range of peak load was less than 3% when the horizontal joint width was reduced from 500 to 300 mm, and the influence of joint width on peak load was not obvious. This shows the precast panel with 300 mm horizontal joint width can meet the load-bearing capacity of the structure. However, in order to explore the minimum horizontal joint width, smaller joint width tests on the precast panel should be conducted for further study.

**Figure 17:**
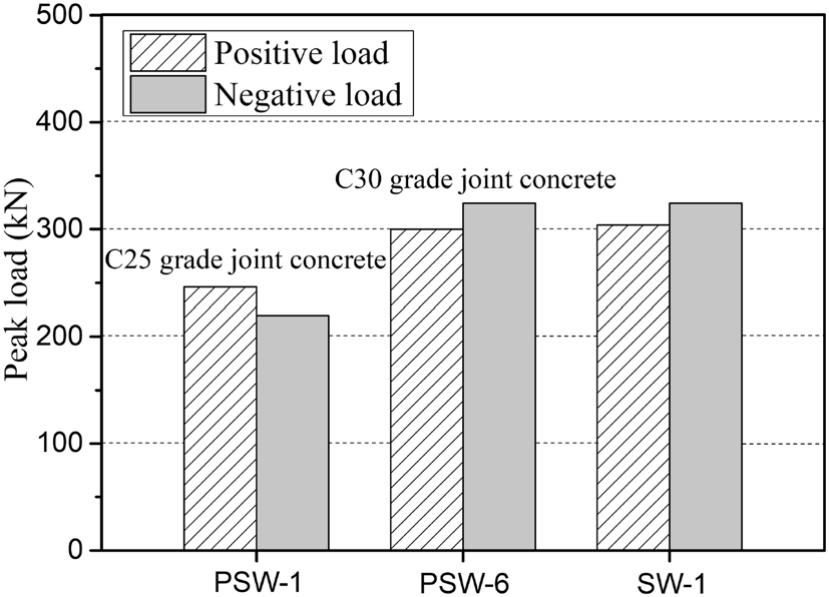
Influence of joint concrete strength on peak load.

As can be seen from Fig. 17, when the concrete strength of the cast-in-place part at the horizontal joint was one grade above that of the upper precast part, the horizontal load-carrying capacity of the precast specimen was significantly enhanced, and the peak load of the precast specimen PSW-6 was close to that of the cast-in-place specimen SW-1 which used the same concrete strength as the precast part of PSW-6. In general, the bottom corner of the concrete panel is a major area where concrete compression damage significantly impacts the structure's final bearing capacity under seismic load^9,30^. In this precast specimen, enough bottom area was reserved for the cast-in-place part by setting the support pillars in the middle of the panel to enhance the horizontal load-carrying capacity. Furthermore, due to the vertical reinforcement connection through U-shaped bar loop overlap, the core concrete within the overlap length range not only underwent compressive stress when the section was in the compression zone but was also subjected to the compression from the bar loop end when it was in the tensile zone (for transferring the tensile stress of vertical reinforcement). Therefore, increasing the compressive strength of the corner concrete has a significant impact on the structural bearing capacity under seismic cyclic load^31,32^.

#### Ductility

Displacement $\Delta$ and displacement angle $\theta$ of each characteristic point are shown in Table 3. The ductility coefficient $\mu$ is the ratio of the ultimate displacement ${\Delta }_{u}$ to yield displacement ${\Delta }_{y}$, i.e., $\mu ={\Delta }_{u}/{\Delta }_{y}$. As can be seen from the Table 3, both peak displacement angle ${\theta }_{p}$(1/102 ~ 1/55) and ultimate displacement angle ${\theta }_{u}$(1/58 ~ 1/38) of all specimens were greater than the limit of the elastic–plastic interlayer displacement angle required by GB 50,011–2010 (the limit of reinforced concrete wall is 1/120), indicating that the precast structure in the test has good deformation capacity and sufficient surplus displacement beyond the scope required by the code. The displacements of each characteristic point of precast specimens were smaller than those of the cast-in-place specimen, but the ductility coefficients μ of the two types of specimens were relatively close, and the maximum difference was less than 8%.

### Stiffness degradation

Under cyclic loading, the stiffness of the specimen gradually degrades due to damage accumulation in the specimen as the cycle number increases. The stiffness degradation of each specimen can be evaluated and compared by computing the secant stiffness ${K}_{i}$ of the specimens under each horizontal loading cycle. The formula for secant stiffness ${K}_{i}$ is given as follows:

2 $$K_{i} = \frac{{\left| { + F_{i} } \right| + \left| { - F_{i} } \right|}}{{\left| { + \Delta_{i} } \right| + \left| { - \Delta_{i} } \right|}}$$

where $+{F}_{i}$ and $-{F}_{i}$ are maximum positive and negative horizontal load under the *i*-th loading cycle, respectively, and $+{\Delta }_{i}$ and $-{\Delta }_{i}$ are the corresponding top displacements of maximum positive and negative load under the *i*-th loading cycle, respectively.

**Figure 18:**
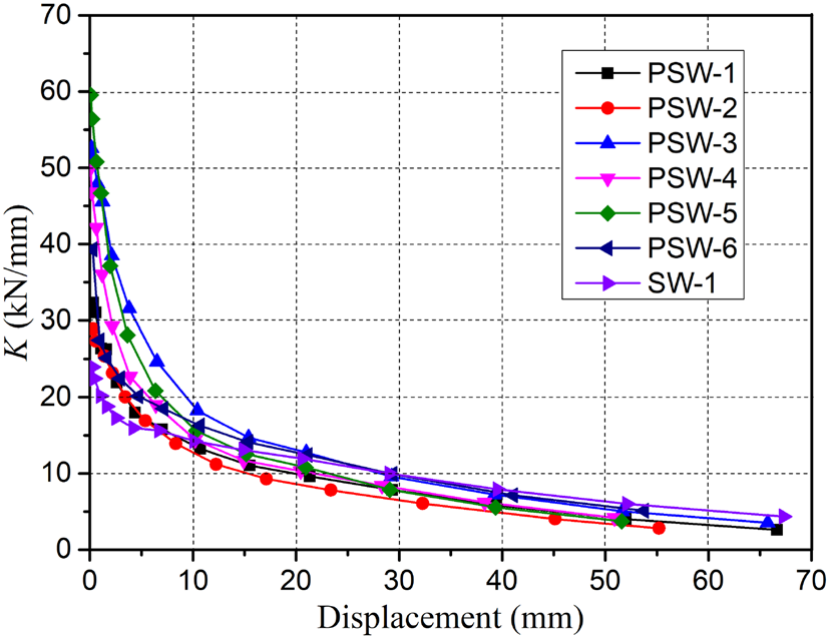
Secant stiffness degradation versus loading displacement.

The secant stiffness degradation trend of all specimens with the increase of loading displacement is shown in Fig. 18. The secant stiffness of all specimens had similar degradation behavior and decreases gradually as the cyclic displacement increased. The secant stiffness of the specimen degrades rapidly due to cracking and damage accumulation at the early loading stage. When the number of the main cracks is no longer increasing, and the loading reaches the yield load, the degradation rate of the secant stiffness is significantly reduced, and the degradation curve shows a gentle downward trend. In light of that, it can be noted from the precast specimens with different influencing factors, the initial stiffness of the precast specimen with a higher axial compression ratio (PSW-3) or smaller joint width (PSW-5) was larger, but the stiffness degradation rate was also faster before reaching the yielding load. The initial stiffness of the precast specimen is generally higher than that of the cast-in-place specimen; however, the stiffness degradation rate is significantly faster than that of cast-in-place specimen due to the cracking at the interface between old and new concrete and the damage accumulation of the joint concrete in the precast specimen. When the yielding load is exceeded, the change in secant stiffness of all specimens follows a similar trend.

### Energy dissipation capacity

**Figure 19:**
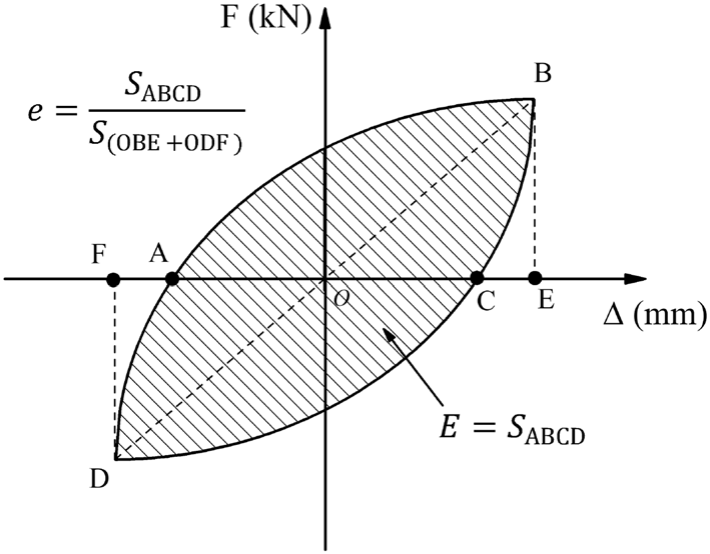
Schematic of dissipated energy *E* and energy dissipation coefficient *e*.

**Figure 20:**
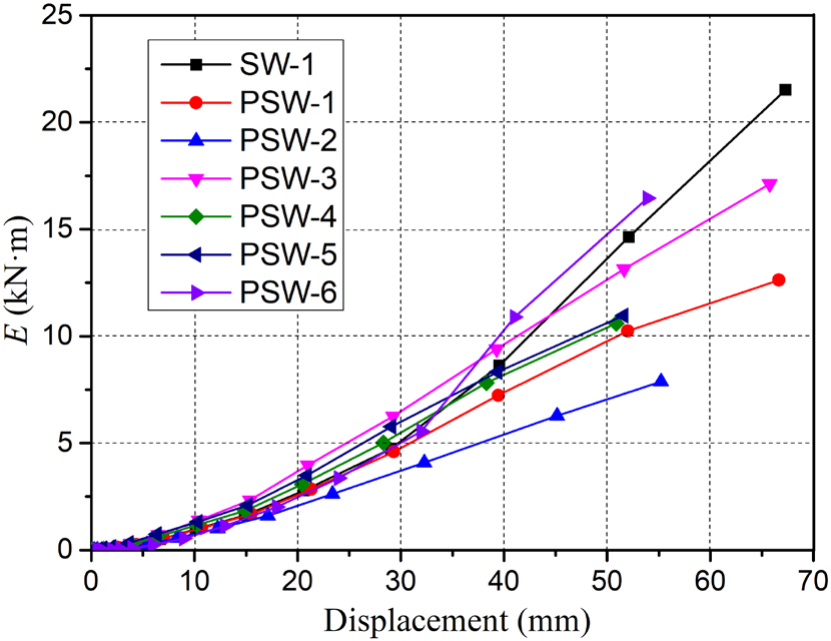
Energy dissipation *E* versus loading displacement.

The dissipated energy *E*_*i*_ and the energy dissipation coefficient *e* can be generally described as the specimens' energy dissipation capacity under cyclic loading. The dissipated energy *E*_*i*_ is calculated using the area enclosed of the load–displacement hysteretic curve in a single cycle, which is the difference between the energy absorbed during loading and the energy released during unloading. The energy dissipation coefficient *e* can further be used to compare the energy dissipation capacity of different specimen types. The expression of the dissipated energy *E*_*i*_ and the energy dissipation coefficient *e* is shown in Fig. 19. The dissipated energy of all specimens increased with the loading displacement, as shown in Fig. 20. The dissipated energy of the cast-in-place specimen SW-1 and the precast specimen PSW-6 with higher joint concrete strength increased rapidly after peak loading. The precast specimens with different joint widths showed similar changes in dissipated energy, and the specimens with higher axial compression ratios exhibited higher dissipated energy. The calculated energy dissipation coefficients of all specimens at each characteristic point are listed in Table 4.

**Table 4:** Energy dissipation coefficient of characteristic points.

Specimen number	Energy dissipation coefficient *e*		
	Cracking point	Yield point	Peak point
SW-1	0.54	0.51	0.87
PSW-1	0.53	0.57	0.75
PSW-2	0.41	0.57	0.66
PSW-3	0.38	0.59	0.79
PSW-4	0.45	0.61	0.71
PSW-5	0.41	0.63	0.72
PSW-6	0.43	0.53	0.91

The energy dissipation coefficient increased with the loading stage. The energy dissipation coefficients of precast specimens at the cracking point were between 0.38 and 0.53, which was slightly lower than that of the cast-in-place specimen. The precast specimen PSW-3 with an axial compression ratio of 0.3 showed the lowest energy dissipation coefficient. At the yield point, the energy dissipation coefficients of precast specimens had little difference, and all precast specimens were higher than those of cast-in-place specimens. When reaching the peak point, the energy dissipation coefficient of SW-1 was higher than that of the standard precast specimen PSW-1; however, the specimen PSW-6 with higher joint concrete strength showed better energy dissipation capacity. During the whole process, the joint width had little influence on the energy dissipation of the precast specimen, and the higher axial compression ratio could slightly increase the energy dissipation of the precast specimen. Also, the energy dissipation capacity of the precast specimen could be significantly improved by increasing the concrete strength of the bottom joint.

## Hysteretic model

### Envelope curve model

**Figure 21:**
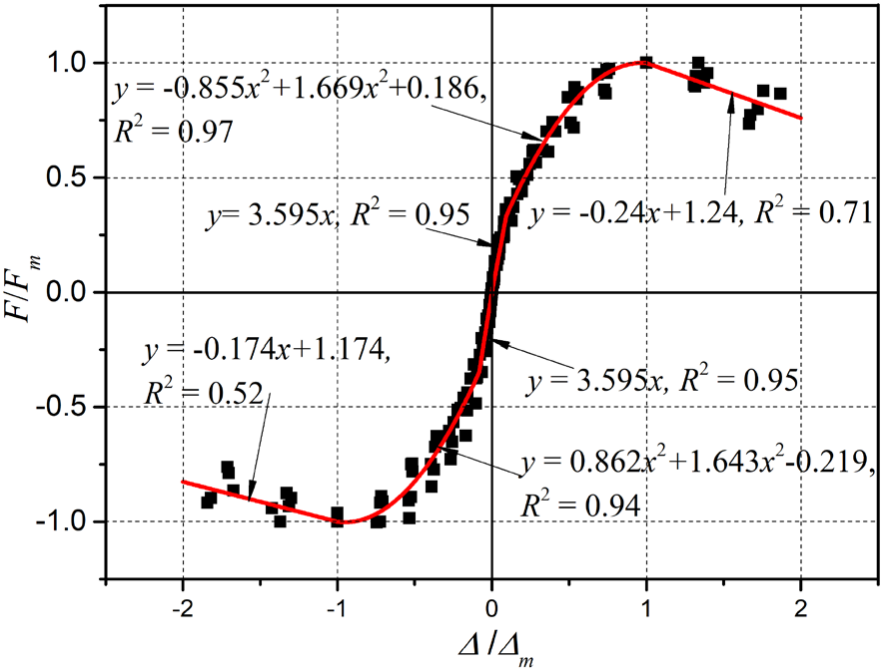
Calculation results of envelope curve model.

A three-segment envelope curve model of precast concrete panels with castellated keys support pillar connections was established according to the characteristics of the envelope curve in Section Envelope curves and characteristic parameters. Considering the influence of axial compression ratio, joint width, and joint concrete strength, all curves were further made dimensionless with the positive and negative peak points $\left( {\left| {\Delta_{p}^{ \pm } } \right|,{ }\left| {F_{p}^{ \pm } } \right|} \right)$ as reference points. The scatter diagram of all dimensionless envelope curves is demonstrated in Fig. 21. The regression equation is represented in a three-segment model based on the envelope curve properties as follows:

3 $$y = \left\{ {\begin{array}{*{20}c} {3.595x,} & {\left( {0 < x \le \frac{{\Delta_{cr}^{ + } }}{{\left| {\Delta_{p}^{ + } } \right|}}} \right)} \\ { - 0.855x^{2} + 1.669x + 0.186,} & {\left( {\frac{{\Delta_{cr}^{ + } }}{{\left| {\Delta_{p}^{ + } } \right|}} < x \le 1} \right)} \\ { - 0.24x + 1.24,} & {(1 < x \le 2) } \\ \end{array} } \right.$$

4 $$y = \left\{ {\begin{array}{*{20}c} {4.235x ,} & {\left( {\frac{{\Delta_{cr}^{ - } }}{{\left| {\Delta_{p}^{ - } } \right|}} \le x < 0} \right)} \\ {0.862x^{2} + 1.643x - 0.219,} & {\left( { - 1 \le x < \frac{{\Delta_{cr}^{ - } }}{{\left| {\Delta_{p}^{ - } } \right|}}} \right) } \\ { - 0.174x - 1.174,} & {(2 \le x < - 1)} \\ \end{array} } \right.$$

where $x$=$\Delta /\left|{\Delta }_{p}^{\pm }\right|$ is the dimensionless independent variable for displacement, $y$=$F/\left|{F}_{p}^{\pm }\right|$ is the dimensionless dependent variable for load; the positive crack displacement ${\Delta }_{cr}^{+}$ = 0.0929 $\left|{\Delta }_{p}^{+}\right|$, the positive initial stiffness ${k}_{0}^{+}=3.595{F}_{p}^{+}/{\Delta }_{p}^{+}$; the negative crack displacement ${\Delta }_{cr}^{-}$ =  − 0.0824 $\left|{\Delta }_{p}^{-}\right|$, the positive initial stiffness ${k}_{0}^{-}=4.235{F}_{p}^{-}/{\Delta }_{p}^{-}$. A comparison of the scatter diagram, the calculated curve obtained using the three-segment envelope curve model, and the coefficient of determination *R*^2^ for the curve is shown in Fig. 21. It can be observed that the calculated results showed good agreement with the dimensionless envelope curve obtained from the test. The model can be used as a reference for static nonlinear analysis of a precast concrete panel system with castellated keys and support pillar connections.

### Stiffness degradation law

**Figure 22:**
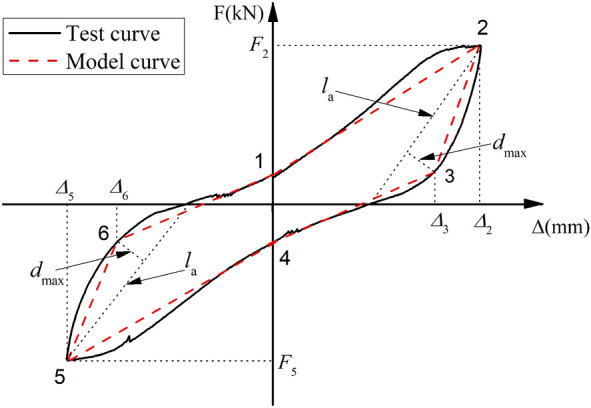
Characteristics of typical single hysteretic loop.

Based on the analysis of the characteristics of the hysteretic curve of the precast panel in Section “Hysteresis curves”, the loading stiffness and unloading stiffness of the hysteretic loop gradually degenerated after the initial elastic segment under the cyclic loading. The unloading part of the hysteretic loop had a larger curvature, and the loop exhibited a pinching phenomenon for a typical single hysteretic loop in the elastic–plastic regime, as shown in Fig. 22. A hysteretic loop could be described by a multi-segment model with six feature points. The intersection points of the loop and the Y-axis in the multi-segment model are points 1 and 4, respectively, while points 2 and 5 are the peak points of the positive and negative hysteretic loops. The auxiliary line *l*_*1*_ is drawn between the peak point and the X-axis intersection of the loop, and the farthest point 3 can be obtained by calculating the furthest distance *d*_*m*_ from the auxiliary line *l*_*a*_ in the unloading Sect. (0 ≤ *F* ≤ *F*_2_) of the positive loop, to better express the large curvature of the unloading part of the hysteretic loop. Similarly, point 6 of the negative loops can be calculated.

**Figure 23:**
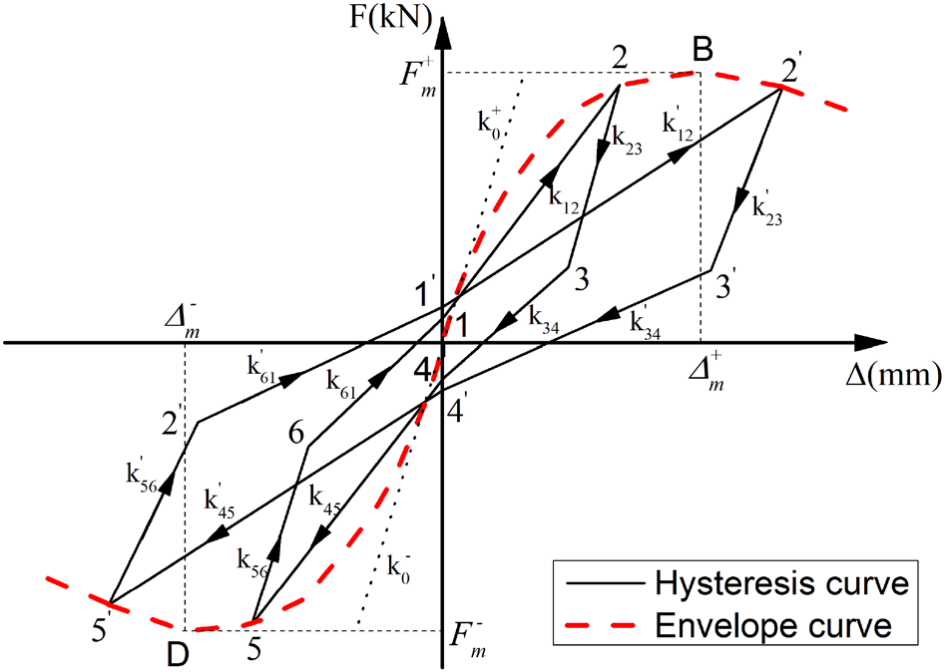
Stiffness degradation law.

The stiffness degradation law of the hysteretic curve is further expressed by the stiffness of each segment degenerating with increasing cyclic displacement in the multi-segment hysteretic loop model. The hysteretic loops $1\sim 6$ and ${1}^{\mathrm{^{\prime}}}\sim {6}^{\mathrm{^{\prime}}}$ under the different loading displacements in the elastic–plastic regime is shown in Fig. 23. It can be observed that the initial stiffness ${k}_{0}^{+}$ in the positive loop gradually degenerated to ${k}_{12}$ and then finally to ${k}_{12}^{\mathrm{^{\prime}}}$ with increasing loading displacement. In the negative loop, the initial stiffness ${k}_{0}^{-}$ also degenerated to ${k}_{45}$ and finally to ${k}_{45}^{\mathrm{^{\prime}}}$. As for the unloading stage of the hysteretic loop, the unloading stiffness ${k}_{23}$ and ${k}_{34}$ of the positive loop respectively degenerated to ${k}_{23}^{\mathrm{^{\prime}}}$ and ${k}_{34}^{\mathrm{^{\prime}}}$ with increasing loading displacement (same for ${k}_{56}$ and ${k}_{61}$ of the negative loop). Thus, the hysteretic curve model of the precast concrete panel's system could be established by analyzing the degradation law of each segment stiffness in the multi-segment hysteretic loop model.

**Figure 24:**
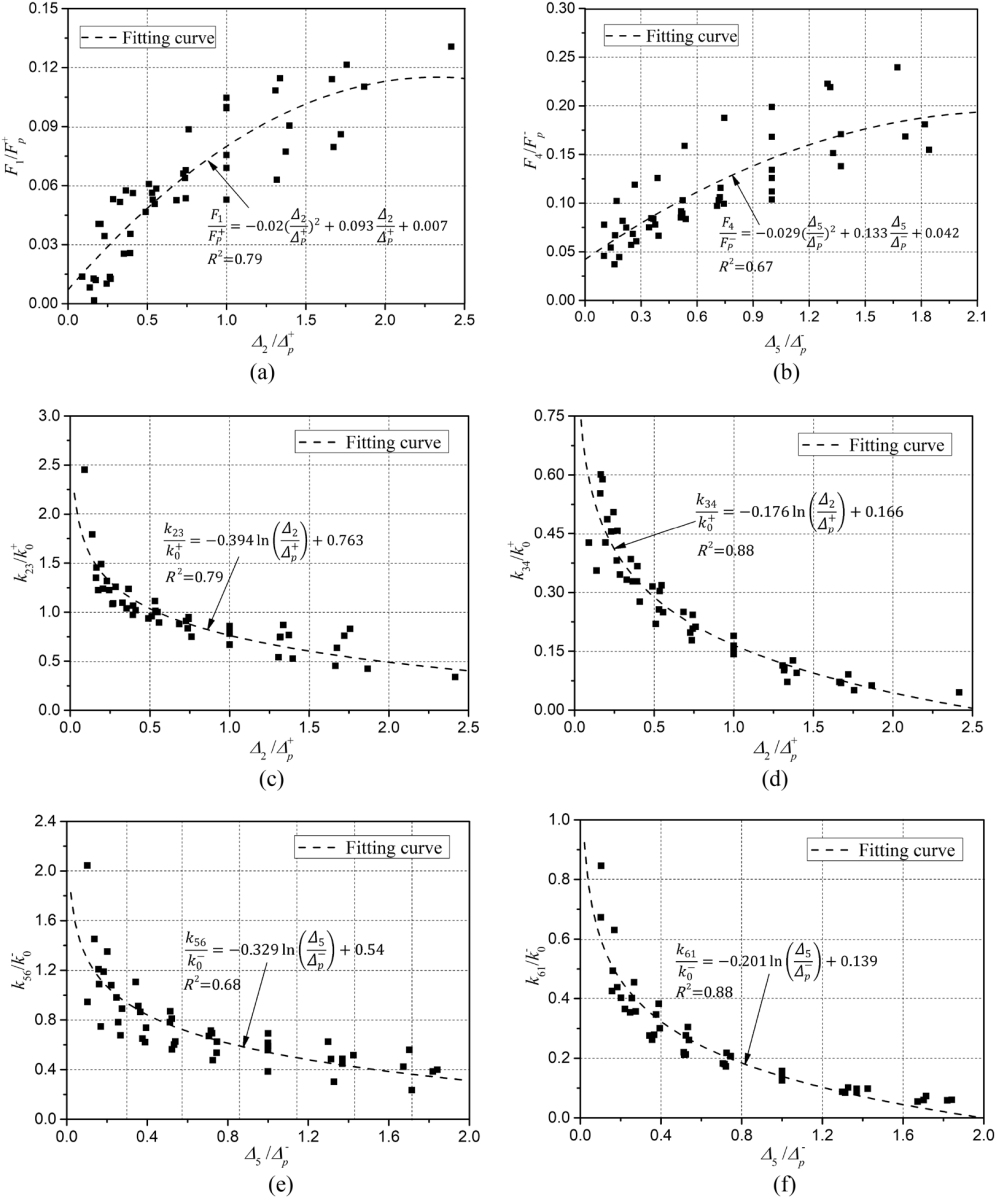
Variation of Stiffness degradation parameters at various stages: (**a**) *F*_1_; (**b**) *F*_4_; (**c**) ${k}_{23}$; (**d**) ${k}_{34}$; (**e**) ${k}_{56}$; (**f**) ${k}_{61}$.

Firstly, point 2 (*Δ*_2_, *F*_2_) and point 5 (*Δ*_5_, *F*_5_), in the positive loading segment 1–2 and the negative loading segment 4–5, could be calculated from the envelope curve model Eqs. (3, 4), where *Δ*_2_ and *Δ*_5_ are the positive and negative loading displacements of this calculated loop, respectively. Subsequently, the test data of each point were made dimensionless with the positive and negative peak points (${\Delta }_{p}^{\pm }, {F}_{p}^{\pm }$) as reference points, and the fitting formula for calculating point 1 (0, *F*_1_) was obtained by using regression analysis of the correlation between point 1 and positive loading displacement *Δ*_2_ as shown in Eq. (5). Similarly, the fitting formula Eq. (6) for calculating point 4 (0, *F*_*4*_) could be obtained. The variation of *F*_1_ and *F*_*4*_ are shown in Fig. 24a, b.

5 $$\frac{{F_{1} }}{{F_{P}^{ + } }} = - 0.02\left( {\frac{{\Delta_{2} }}{{\Delta_{P}^{ + } }}} \right)^{2} + 0.093\frac{{\Delta_{2} }}{{\Delta_{P}^{ + } }} + 0.007$$

6 $$\frac{{F_{4} }}{{F_{P}^{ - } }} = - 0.029\left( {\frac{{\Delta_{5} }}{{\Delta_{P}^{ - } }}} \right)^{2} + 0.133\frac{{\Delta_{5} }}{{\Delta_{P}^{ - } }} + 0.042$$

Secondly, the degradation law of the dimensionless unloading stiffness ${k}_{23}/{k}_{0}^{+}$ and ${k}_{34}/{k}_{0}^{+}$ with the change of loading displacement ${\Delta }_{2}/{\Delta }_{p}^{+}$ was calculated through regression analysis, as shown in Fig. 24c, d. The fitting formulas are shown in Eq. (7 and 8).

7 $$\frac{{k_{23} }}{{k_{0}^{ + } }} = - 0.394\ln \left( {\frac{{\Delta_{2} }}{{\Delta_{p}^{ + } }}} \right) + 0.763$$

8 $$\frac{{k_{34} }}{{k_{0}^{ + } }} = - 0.176\ln \left( {\frac{{\Delta_{2} }}{{\Delta_{p}^{ + } }}} \right) + 0.166$$

Finally, the intersection of the unloading segments 2–3 and segments 3–4 could be calculated to obtain the coordinates of point 3 (${\Delta }_{3}$, ${F}_{3}$) according to the unloading stiffness ${k}_{23}$, ${k}_{34}$ and the coordinates of point 2 and point 4, as shown in Eq. (9, 10).

9 $$\Delta_{3} = \frac{{F_{4} - F_{2} + k_{23} \Delta_{2} }}{{k_{23} - k_{34} }}$$

10 $$F_{3} = k_{34} \frac{{F_{4} - F_{2} + k_{23} \Delta_{2} }}{{k_{23} - k_{34} }} + F_{4}$$

Similarly, the unloading stiffness ${k}_{56}$, ${k}_{61}$ in the negative unloading segment 5–6-1 and the coordinates of point 6 (${\Delta }_{6}$, ${F}_{6}$) could be obtained. The stiffness degradation laws of the unloading stiffness ${k}_{56}$ and ${k}_{61}$ are shown in Fig. 24e,f, and the fitting formulas are shown in Eqs. (11) and (12). The coordinates of point 6 are calculated in Eqs. (13) and (14).

11 $$\frac{{k_{56} }}{{k_{0}^{ - } }} = - 0.329\ln \left( {\frac{{\Delta_{5} }}{{\Delta_{p}^{ - } }}} \right) + 0.54$$

12 $$\frac{{k_{61} }}{{k_{0}^{ - } }} = - 0.201\ln \left( {\frac{{\Delta_{5} }}{{\Delta_{p}^{ - } }}} \right) + 0.139$$

13 $$\Delta_{6} = \frac{{F_{1} - F_{5} + k_{56} \delta_{5} }}{{k_{56} - k_{61} }}$$

14 $$F_{6} = k_{61} \frac{{F_{1} - F_{5} + k_{56} \delta_{5} }}{{k_{56} - k_{61} }} + F_{1}$$

### Hysteretic model calculation results

**Figure 25:**
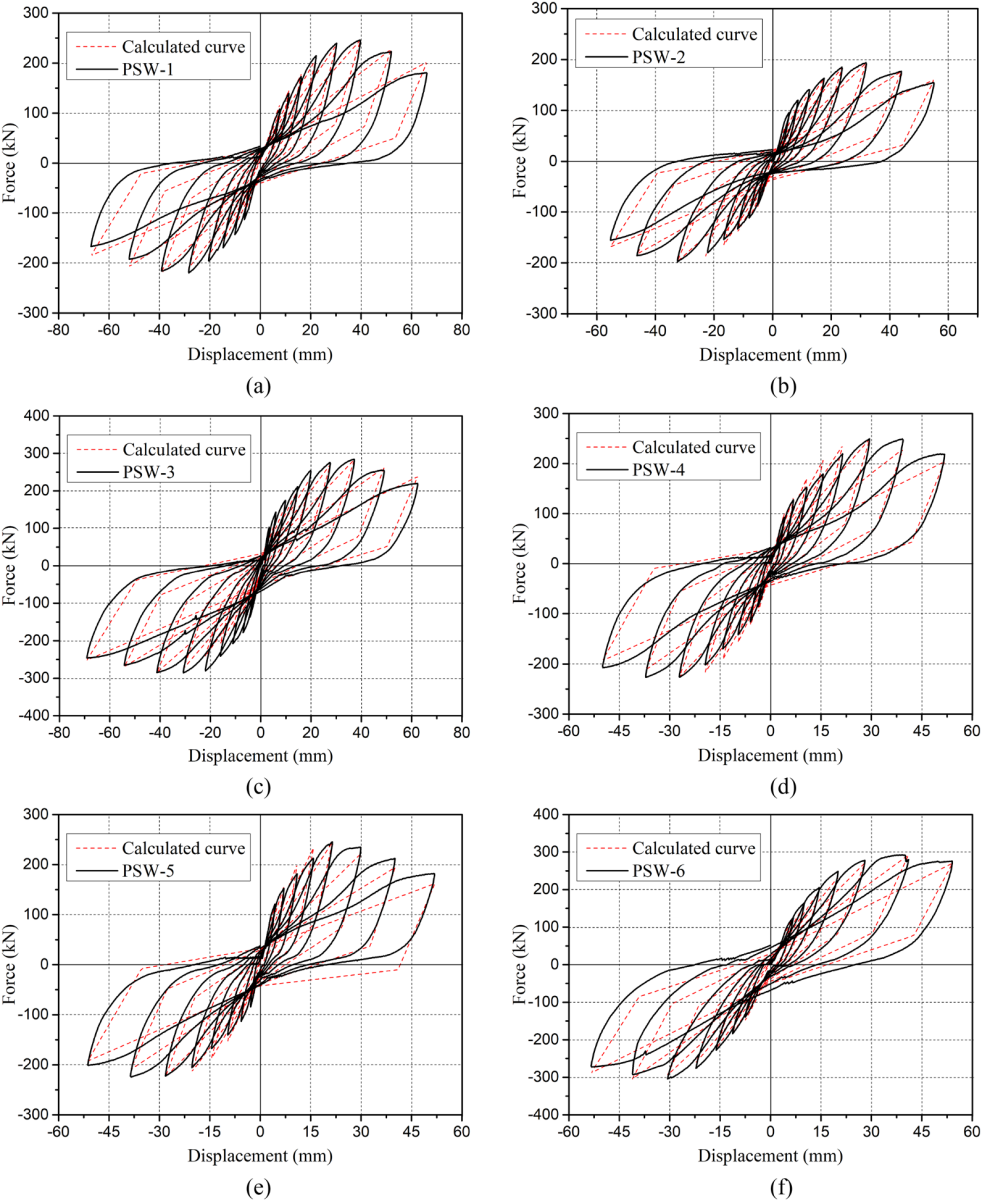
Comparison of hysteretic model calculation and test results.

The envelope curve model and stiffness degradation law comprise the hysteretic curve model of the precast concrete panel. When the loading displacement has not reached the cracking displacement under cyclic loading, the precast specimen is in elastic behavior, and the initial stiffness expresses the current hysteretic curve according to the three-segment envelope curve. When the precast specimen reaches the elastic–plastic behavior, the stiffness degradation law can be used to calculate the feature point coordinates of the hysteresis loop in the current loading displacement. Thus, the hysteretic loops of the precast specimen under different loading displacements can be predicted. A comparison of the test curve and the calculated curve using the hysteretic curve model is shown in Fig. 25. It can be observed that results obtained from the hysteretic curve model showed good agreement with test results, and the model can well reflect the hysteretic behavior of the precast concrete panels with castellated keys support pillar connections.

## Conclusions

The experimental evaluation of the seismic performance of precast concrete panels with castellated keys support pillar connections was presented in this paper. Six full-size precast concrete wall panels and one cast-in-place wall panel were subjected to in-plane cyclic loading to investigate a number of seismic response indices, such as fracture patterns, failure modes, bearing capacity, ductility, stiffness deterioration, and energy dissipation capacity. The following are the primary findings that can be derived from the experimental results and analysis:

(1) All specimens exhibited that similar failure modes are compression and bending under in-plane cyclic loading. The initial horizontal cracks of precast specimens appeared first at the upper interface of the cast-in-place area in the horizontal joint, and its cracking load was less than that of the cast-in-place specimen. But increasing the strength of joint concrete can effectively delay the appearance of cracks in the interface between old and new concrete. Additionally, as the core concrete in the U-shaped bar overlap area is subjected to compressive stress, regardless of whether the horizontal joint section is in the compression zone or the tension zone, the joint concrete strength is crucial to the bearing capacity of the precast structure.

(2) The load–displacement curve of all specimens changed linearly in the initial stage, and the slope of the ascending part of the curve gradually decreased after the panel cracking. When the horizontal load reaches the peak, the precast specimen's pinching phenomenon is more pronounced than the cast-in-place specimen. But the descending part of the hysteretic curve of the precast specimen was fuller. Furthermore, increasing the strength of the joint concrete could significantly reduce the pinching phenomena.

(3) The main characteristic points were defined according to the three-segment envelope curves. The cracking load and the peak load of the precast specimens increased with increasing the axial compression ratio. Appropriately increasing the axial compression ratio improved the horizontal load-carrying capacity of the structure. The joint width in the test range had little effect on the peak load of the precast panel. The higher joint concrete strength can obviously increase the cracking load and effectively enhance horizontal load-carrying capacity of the precast structure.

(4) The peak displacement angle and ultimate displacement angle of the precast specimens were greater than the limit of the elastic–plastic interlayer displacement angle required by GB 50,011–2010, and the ductility coefficient of the precast specimens was close to that of the cast-in-place specimen. This indicated that the precast structure in the test had good deformation capacity.

(5) The secant stiffness of all specimens declined rapidly due to cracking and damage accumulation at the early loading stage. The initial stiffness of the precast specimen was generally higher than that of the cast-in-place specimen; however, the stiffness degradation rate was significantly faster than that of cast-in-place specimens. When the yielding load is exceeded, the change in secant stiffness of all specimens follows a similar trend.

(6) The energy dissipation coefficient of the precast specimen was greater than that of the cast-in-place specimen at the yield point but less than that of the cast-in-place specimen at the peak point. Throughout the test, the energy dissipation capacity of the precast specimens was close to that of the cast-in-place specimen.

(7) Based on the characteristics of the three-segment envelope curves model and the stiffness degradation law, a multi-segment hysteretic loop model of precast concrete panels with castellated keys support pillar connections was formulated. The calculation formulas of the envelope curves model and the stiffness degradation law were gained by regression statistics on the dimensionless test data. The forecast results from the envelope curves model and the hysteretic loop model showed good agreement with the test results. The model can be used as a reference to analyze the seismic performance of precast concrete panels with castellated key support pillar connections.

## Data Availability

The datasets used and/or analysed during the current study available from the corresponding author on reasonable request.

## References

[1] PCI Industry Handbook Committee. PCI design handbook: Precast and prestressed concrete In: *Prestressed Concrete Inst*, 7th ed. (2010).

[2] Yu SS, Liu YF, Wang DJ (2021). Review of thermal and environmental performance of prefabricated buildings: Implications to emission reductions in China. Renew. Sust. Energ. Rev..

[3] Wu G, Feng DC (2018). Research progress on fundamental performance of precast concrete frame beam-to-column connections. J. Build Struc..

[4] Seifi P, Henry RS, Ingham JM (2019). In-plane cyclic testing of precast concrete wall panels with grouted metal duct base connections. Eng. Struct..

[5] Nye TK, Pantelides CP, Burningham CA (2018). Unidirectional GFRP composite connections between precast concrete wall panels under simulated seismic loads. Compos. Struct..

[6] Ujianto M, Ali AZM, Solikin M (2019). Structural behavior of precast concrete wall panels due to dynamic load: A review. AIP Conf. Proc..

[7] Singhal S, Chourasia A, Chellappa S, Parashar J (2019). Precast reinforced concrete shear walls: State of the art review. Struct. Concrete.

[8] Pimanmas A, Yooprasertchai E, Wiwatrojanagul P (2019). Cyclic loading test of precast concrete load-bearing walls designed for gravitational loading. Mag. Cconcrete Res..

[9] Becker JM, Llorente C, Mueller P (1980). Seismic response of precast concrete walls. Earthq. Eng. Struct. Dyn..

[10] Soudki KA, West JS, Rizkalla SH, Blackett B (1996). Horizontal connections for precast concrete shear wall panels under cyclic shear loading. PCI J..

[11] Xiao TL, Sheng JC, Chen Y, Chen BY (2019). Research progress on bolting connection of prefabricated concrete shear wall. IOP Conf. Ser. Mater. Sci. Eng..

[12] Han QH, Wang DY, Zhang YS, Tao WJ, Zhu Y (2020). Experimental investigation and simplified stiffness degradation model of precast concrete shear wall with steel connectors. Eng Struct.

[13] Sun CF, Liang ST, Zhu XJ (2020). Experi mental study and numerical simulation of precast shear wall with rabbet-unbonded horizontal connection. Int. J. Concr. Struct. M..

[14] Li XH, Wu G, Kurama YC, Cui HR (2020). Experimental comparisons of repairable precast concrete shear walls with a monolithic cast-in-place wall. Eng. Struct..

[15] Xiong F, Malla P, Cai GC, Larbi AS, Zhong YC (2020). Numerical analysis of precast concrete shear walls with horizontal bolted joints under seismic loads. J. Earthq. Eng..

[16] Psycharis IN, Kalyviotis IM, Mouzakis HP (2018). Experimental investigation of the response of precast concrete cladding panels with integrated connections under monotonic and cyclic loading. Eng. Struct..

[17] Gu Q, Dong G, Wang X, Jiang HB, Peng SM (2019). Research on pseudo-static cyclic tests of precast concrete shear walls with vertical rebar lapping in grout-filled constrained hole. Eng. Struct..

[18] Wu M, Liu X, Liu HT, Du XL (2020). Seismic performance of precast short-leg shear wall using a grouting sleeve connection. Eng. Struct..

[19] Xu F, Wang K, Wang SG (2018). Experimental bond behavior of deformed rebars in half-grouted sleeve connections with insufficient grouting defect. Constr. Build Mater..

[20] Reichenbach S, Kromoser B (2021). State of practice of automation in precast concrete production. J Build Eng.

[21] Kurama YC, Sritharan S, Fleischman RB, Restrepo JI, Henry RS, Cleland NM (2018). Seismic-resistant precast concrete structures: State of the art. J. Struct. Eng..

[22] Madireddy H, Naganathan S, Mahalingam B (2022). Sustainable practices and innovations in civil engineering. ICSPICE.

[23] Padil KH, Bady HMA, Saim AA (2014). Ultimate shear capacity and failure of shear key connection in concrete construction. Malays. J. Civ. Eng..

[24] GB50011–2010. *Code for Design of Concrete Structures*. China Architecture & Building Press (in chinese) (2010).

[25] JGJ 3–2010. *Technical Specification for Concrete Structures of Tall Building*. China Architecture & Building Press (in chinese) (2010).

[26] GB/T 50081–2019. *Standard for Test Methods of Concrete Physical and Mechanical Properties*. China Architecture & Building Press (in chinese) (2019).

[27] GB/T 228.1–2010. *Metallic Materials-Tensile Testing-Part 1: Method of Test at Room Temperature*. China Architecture & Building Press (in chinese) 2010.

[28] JGJ/T 101–2015. *Specification for Seismic Test of Buildings*. China Architecture & Building Press (in chinese) (2015).

[29] Park R (1989). Evaluation of ductility of structures and structural assemblages from laboratory testing. Bull. N. Z. Natl. Soc. Earthq. Eng..

[30] FEMA 273. *NEHRP Guidelines for the Seismic Rehabilitation of Buildings (FEMA 273)*, Federal Emergency Management Agency (1997).

[31] Basit S, Maki T, Mutsuyoshi H, Ishihara Y, Tajima H (2020). Influence of reinforcement arrangement details on mechanical behavior of precast concrete barrier with loop connection. Structures.

[32] Joergensen HB, Hoang LC (2020). Tests and limit analysis of loop connections between precast concrete elements loaded in tension. Eng. Struct..

